# Identifying and assessing views among physically-active adult gym members in Israel on dietary supplements

**DOI:** 10.1186/s12970-017-0194-7

**Published:** 2017-09-21

**Authors:** Inbal Druker, Anat Gesser-Edelsburg

**Affiliations:** 1 School of Public Health, University of Haifa, 199 Aba Khoushy Ave., Mount Carmel, 3498838 Haifa, Israel; 2 The Zinman College of Physical Education and Sport Sciences, Wingate Institute, 42902 Netanya, Israel; 3 Health Promotion Program, School of Public Health, Health and Risk Communication Research Center, University of Haifa, 199 Aba Khoushy Ave., Mount Carmel, 3498838 Haifa, Israel

**Keywords:** Sports dietary supplements, Risk perception, Health, Authority, Trainer, Qualitative research

## Abstract

**Background:**

Sports dietary supplements are available for sale in public places including sports clubs. Although there is uncertainty regarding their safety, many gym members who regularly work out consume them. The present study aimed to identify the approaches and perspectives of the public who work out in gyms and take dietary supplements. It examined how professionals view sports dietary supplement consumption, and how they communicate this issue to gym members. The literature discusses the prevalence of SDS use among athletes, but rarely discusses or compares between the risk perceptions of gym members, trainers, and dietitians, who represent the physically-active general public, regarding SDS.

**Methods:**

We conducted constructivist qualitative research in semi-structured one-on-one interviews (*n* = 34). We held in-depth interviews (*n* = 20) with a heterogeneous population of adult gym members who take dietary supplements, and (*n* = 14) with dietitians and fitness trainers.

**Results:**

The main finding was a gap in risk perception of dietary supplement use between dietitians, gym members and fitness trainers. There was low risk perception among dietary supplements consumers. Trainers believed that benefits of supplement consumption exceeded risk, and therefore they did not convey a message to their clients about risk. In contrast, dietitians interviewed for this study renounced general use of sports dietary supplements and doubted whether trainers had proper nutritional knowledge to support it.

**Conclusion:**

Lack of awareness of risks suggests that there is a need for communication on this issue. We recommend that professionals (physicians and dietitians) be present in sports clubs that sell such products in an uncontrolled way.

## Background

### Prevalence of sports dietary supplement use

The prevalence of using sports dietary supplements (SDS) has increased over the last 30 years among adults in the United States. Approximately half of the adult population in the United States reports using one or more dietary supplement [1]. The prevalence of using sports dietary supplements among gymnasium users in Riyadh, Saudi Arabia in 2015 was 37.8% [2] and in gyms in Lebanon the intake of dietary supplements was observed in 38% of the study participants [3].

In Israel, according to data which were published in the Israeli National Health and Nutrition Survey for the years 1999–2001, 21.5% of the people who were sampled reported taking nutrition supplements [4]. Previous studies on SDS and their effectiveness indicate that some may have side effects and others do for certain. Therefore, they must be used with caution [5]. Nevertheless, the use of SDS among gym members is on the increase.

### Reasons for increased use of SDS among gym members

Many gym goers, who are eager to add muscle bulk, use SDS to maintain a low-fat, thin figure for athletic and esthetic purposes. This is called “body capital” and can have extreme ramifications [6]. A preoccupation with physical appearance is a factor that increases the likelihood of using these products [7, 8]. Other reasons for taking SDS include a combination of social and psychological factors, knowledge and financial conditions [1, 9–13] as well as media influence. The theory of planned behavior assumes that an individual would tend to take SDS if their action involves a combination of social norms with a belief that the action is controlled and made by choice [10]. According to the literature, the consumers of supplements want to maintain health and prevent nutritional deficits, increase their energy level, speed up recovery after physical activity, and boost their immune systems [12]. People tend to overestimate their physiological needs [14], and even when they know that supplements are unnecessary, they may consume them [15], even if they are ineffective [16].

### The risk of taking sports nutrition supplements

Studies indicate that some supplements have been found to contain additional substances that might have side effects, such as caffeine, which might cause anxiety, sleeping disorders, and such cardiovascular problems as high blood pressure and physiological symptoms of emotional turmoil [17]. There is growing evidence about a poor quality of sports nutrition supplements based on their manufacturing and packing processes [18]. The most common problem is the presence of allergens in a product, which are not reported by the manufacturer. Additionally, there is concern about microbiological infection or infection caused by stimulating substances, estrogenic ingredients, variables, and anabolic substances [19, 20]. The presence of estrogenic substances might cause significant health problems when they go beyond the recommended dosage, and this is often the case in sports nutrition supplements [21]. DMAA is an additional substance that is added to nutrition supplements and is known for its ability to lead to weight loss, body contouring, and increased heart rate. It might cause heart problems (particularly sudden heart death), severe liver damage, and liver failure [19]; and melamine, which is added to 47% of the researched nutrition supplements, is a source of nitrogen from a non-protein source and might cause kidney stones [22].

### Loopholes in the regulation

The FDA regulates dietary supplements in the United States, including both the final product and its ingredients. The FDA acts under the Dietary Supplement Health and Education Act of 1994 (DSHEA). Companies are responsible for labeling their products and assessing their safety before marketing them, ensuring that they meet DSHEA and FDA regulations [23].

The loophole in the control process is that although manufacturers are required to register their supplements through the FDA, they are not required to receive approval before producing or selling the product. Manufacturers are not required to test the ingredients or new supplements in clinical trials. DSHEA allows the FDA to stop a company from manufacturing a particular dietary supplement only when the FDA proves that it causes damage. There is no need for a prescription for dietary supplements, so there is no controlled system through which one can report negative reactions and side effects.

In Israel, dietary supplements are regulated according to regulations that apply to food, which require labeling to be “true and not misleading.” The Health Ministry can stop the marketing of a supplement that claims to have “healing qualities” only if it is hazardous to public health [24]. Due to the current regulation on dietary supplements, many products that have never been tested are sold over the counter [25].

SDS have been sold OTC in gyms too. Some studies have shown that SDS users take them without consulting anyone [26]. With regard to competitive professional sports, studies have shown that men consult with the salespeople who work at stores selling SDS. Women tend to consult with family members. Neither men nor women tend to consult with professionals [27, 28]. There are two issues not addressed by previous studies that this study addresses. First, studies have not addressed the views of professionals (dietitians and trainers) on SDS consumption. Second, they have also not addressed how gym members view different types of professionals in the context of their views on SDS consumption.

The present study seeks to fill this gap and ascertain risk perceptions of different target audiences, in order to examine whether there is a gap between perceptions among nutrition specialists and the risk perception among the community of trainees and trainers.

### Communicating SDS risk

SDS involve uncertainty, and therefore there is a need to communicate risks to the public and convey a message that every active substance might have benefits but can also have adverse effects on health [29]. The public’s risk perception is influenced by personal, psychological, environmental and social factors [30, 31]. In order to communicate the risk, there is a need to take the risk communication approach to create an interactive process of exchanging information and opinions among individuals, groups and institutes. The public responds and becomes influenced not only by information about the risk, but also by how the risk is communicated. The public’s assessment of risk is based on criteria not necessarily obvious to experts [32]. This insight encourages the dialogue approach in risk communication, which sees the public as an active part of the process and not a passive one [33].

According to Sandman’s risk communication model [34], risk perception is comprised of the hazard level (risk estimation) and outrage level (the emotions the risk stimulates). If the hazard level and outrage level are similar among the experts and the target audience, the risk may be communicated very effectively, but if they are different, this can lead to controversy. According to Sandman, people associate low risk with issues towards which they have positive attitudes, and vice versa, regardless of the proved risk. In order to communicate the risk successfully, one should consider the public’s feelings about the risk and make efforts to minimize the gap between the estimation of the risk among the experts and the public’s risk perception.

Sports organizations such as ACSM, IOC and ISSN tend to communicate the risk by providing information about the supplements and classifying them into four categories: 1. Apparently effective, 2. Possibly effective, 3. Uncertainty regarding effectiveness, 4. Supplements that seem ineffective and possibly dangerous [20]. According to the Academy of Nutrition and Dietetics (formerly the American Dietetic Association), supplements should be used with caution, and only after careful evaluation of the product for safety, efficacy, potency, and whether or not it is a banned or illegal substance [35]. However, sports organizations do not consider public risk perception. Research indicates that risk perception among the population is low because SDS are perceived as dietary products, and as such, as healthy [36].

The present study seeks to fill this gap and ascertain risk perceptions of different audiences, in order to examine whether there is a gap between perceptions of SDS among nutrition specialists and among the community of gym goers and trainers. This study seeks to understand the perspectives of adults who regularly work out in gyms in Israel versus the approaches and perspectives of professionals regarding SDS consumption, and the risk communication by those considered authorities by gym goers.

## Methods

In order to ascertain approaches and perspectives regarding SDS use, a qualitative constructivist study was designed [25]. Data were collected from a heterogeneous sample of gym members during the years 2014–2015 at a central gym in Israel.

### The research population

The research population consisted of 34 participants that included ten men and ten women, trainees in the sports club in the central area of Israel aged between 18 and 50 and who take sports nutrition supplements.

All of the trainees who were interviewed took supplements. Additionally, we held interviews with 13 professionals, including dietitians and fitness trainers who work in sports clubs all over Israel, and interact in their workplace with trainees who take sports nutrition supplements. The data and socio-demographic characteristics of the participants are listed in Table 1.

**Table 1:** Data and socio-demographic characteristics of the participants (trainees, trainers and dietitians)

Trainees					
No.	Trainee	Age	Gender	Background	Weekly frequency of training
1	A	40	Male	Professor of immunology	Runs outdoors daily and goes to the gym before work.
2	H	27	Male	A former broker	Works out regularly at high intensity.
3	I	26	Male	A student and fitness trainer	Works out at the gym regularly
4	I	50	Male	An accountant	Works out three times/week at the gym +rides a road bike
5	M	29	Male	Security/protection	Works out regularly at the gym + runs outdoors
6	D	21	Male	A lifeguard	Works out approximately three times a week + swims
7	T	29	Male	Student of veterinary medicine	Works out 3–4 times/week
8	B	26	Male	Student	Works out 3–4 times/ week
9	E	37	Male	A PhD student (formerly in high tech)	Works out regularly at the gym
10	I	27	Male	Student	Works out 3–4 times/week at the gym
11	M	20	Female	Soldier in active duty.	Works out intensively on a daily basis. Practices ju jitsu.
12	S	23	Female	MA student from Singapore	Works out at the gym on a weekly basis
13	M	29	Female	A rugby player and trainer	Plays rugby +works out at the gym
14	M	29	Female	A PhD student	Swims +attends classes at the gym
15	M	34	Female	Clinical psychologist	Combines studio classes with gym workouts
16	B	21	Female	Student from the USA	Works out regularly
17	H	40	Female	Learning to be a fitness trainer	Indoor cycling +gym training +outdoor cycling +running daily
18	I	40	Female	A judo wrestler	Six training sessions a week and cross fitness training
19	T	32	Female	High tech	Works out at the gym three times/week
20	D	43	Female	Works at a bank	Works out at the gym three times/week
Trainers					
No.	Trainee	Age	Gender	Background	
1	V	30	Male	Trainer, who practices a professional sport	
2	R	26	Female	Trainer from the Christian Arab sector	
3	A	27	Female	Trainer and body builder	
4	G	28	Male	Trainer	
5	A	30	Male	Trainer	
Dietitians					
No.	Dietitians	Age	Gender	Background	
1	I	45	Female	Clinical dietitian	
2	G	30	Male	Clinical dietitian and sports dietitian	
3	G	40	Male	Clinical dietitian with a BA in physical education	
4	S	43	Male	Clinical dietitian and sports dietitian	
5	A	41	Male	Clinical dietitian and sports dietitian, and food technologist	
6	H	32	Male	Clinical dietitian and sports dietitian	
7	N	40	Male	Clinical dietitian, lecturer and fitness trainer	
8	M	38	Female	Clinical dietitian, former athlete and fitness trainer	
9	R	35	Female	Clinical dietitian, former athlete	

### Research tools

The study was conducted with semi-structured 45-min-long in-depth interviews with gym members, fitness trainers and dietitians. Interview protocol was constructed based on a broad literature review about the prevalence of dietary supplements in general and of sports dietary supplements in particular, relating to key terms from the field. A description is presented in Table 2.

**Table 2:** The semi-structured in-depth interviews protocol

Theme	Questions
Risk perception and risk communication	1. Some people say that taking dietary supplements in different dosages might be effective, but if they are taken beyond these dosages they might have side effects. What is your opinion on this issue? 2. In your opinion, what is the benefit versus the risk of taking dietary supplements? What do you think are the benefits, and do the benefits exceed the risk? 3. For trainers only - As a trainer, do you warn your clients about possible risks?
The source of information and its reliability	1. What sources of information do you use to learn about dietary supplements? 2. Do you think the information is reliable?
The impact of environmental norms	1. Are there any dietary supplements in the gym where you work out? Please describe where they are located. 2. What do you think about it? 3. In your opinion, how does the presence of those supplements at the gym affect the gym members? 4. Does someone in your community (friends/gym members/ family members) use sports dietary supplements? 5. Who assists and directs them as to what kind of supplements they should take and what they are for? 6. Do you know about the consumption of supplements among trainers?
The authority status in the gym members’ point of view	1. In your opinion, which of the following agents (a physician /trainer /dietitian) influences a gym member, and in what order of importance? 2. If there was a case in which a trainer recommended one thing and a physician recommended another, to whom would you listen? 3. If a dietitian and a trainer recommended two different things, to whom would you listen? Why?

### Sample and data collection

In this study, we chose a representative sample that focused on informants who best represented the population. Heterogeneous gym members, including men and women of different ages, were chosen by heterogeneity sampling [37].

### The research procedure

The participants were contacted at a gym in the central area of Israel. One of the researchers introduced the study and its purposes and asked them whether they took supplements. If the respondent answered yes, the interviewer asked for their consent to be interviewed. The meetings were scheduled over the phone. The interviews were held by one of the researchers, in person, in the dietitian’s room in the sports club, at the gym itself, at the interviewees’ homes, or over the phone, for 40–50 min. The trainers were from the same sports club in the central area of Israel and a similar process took place with them. Dietitians were reached from other sports clubs all over Israel. They were approached through dietitian colleagues or through a forum for dietitians on Facebook. In this case the interviews were conducted in a quiet place that was agreed upon by the dietitian interviewee and the interviewer.

### The sampling method

In this study we made a purposeful sample that focused on choosing informants who best represented the population from which they were chosen and who were most capable of teaching us about the researched phenomenon. Different types of trainees, including men and women of different ages, were chosen by heterogeneity sampling (30).

### Quality control and data handling

In order to validate the study, the findings of this study were compared with findings of previous studies. The comparison raised similar conclusions and explained the researched phenomenon [38]. The recordings of the interviews and the transcripts were saved [39]. All the documents were saved, ranging from the raw stage of data collection to the final research findings and conclusions [40]. The text was written as a rich description that included information about the context, quotes from informants, and an open conceptual discussion [41].

For study dependability, we used a semi-structured questionnaire written for the interview and examined by the researchers. After conducting a pilot interview, the questions were edited and revised according to our pilot study. Each interview was recorded and transcribed after the meeting. After recording the interviews, a proof-listening was made for each interview to follow how things were said and how the interview was transcribed. These checks helped us to identify possible mistakes in the data encoding process. The documents were saved at every stage. We interviewed until “saturation point” [42]. New participants did not produce new data in the interviews [40].

### Analysis

The data which were gathered from the interviews were analyzed according to the thematic analysis method [43]. Every interview was analyzed separately, and during this stage themes were constructed. In the second stage, some of the themes were subdivided and others were divided into more specific categories. In each category, we made a reference that presented the approaches and perspectives of the three different sub-groups (trainers, gym members and dietitians). In the third stage we came up with certain themes that integrated the categories, in order to get a clear picture of the categories [44]. In the third stage the three sub-groups were integrated while constructing categories and comparing all the groups.

## Results

The main themes that arose from the interviews are as follows: (1) Risk perception of dietary supplement use and communication of information (2) Sources of information and reliability (3) Social and environmental norms (4) Influence of authority.

### Risk perception of dietary supplement use and communication of information

#### Risk perception among gym members

When gym members were asked, "Do you know of any side effects?" they replied that “protein powder supplements” are not perceived as a supplement with side effects, particularly if they are taken at the recommended dosage as specified on the container. There are other dietary supplements, such as fat burners, which have serious side effects, and whoever experienced them stopped taking them. Although some of the interviewees knew that they might experience side effects, the risk was still expressed as possible but not certain.

*"I know that protein supplements can cause stomach pains and gas, and I think I've heard that creatine might do something but I can't remember what."* (M., a female gym member)

#### Risk perception among trainers

When trainers were asked about side effects, some of the responses were positive, stating that there might indeed be side effects, but most were unaware of the possibility of risk due to the side effects. Some trainers claimed not to have knowledge on the subject and that they had never experienced side effects. Many claimed that the risk exists only after excessive consumption.

When trainers addressed benefit versus risk, they pointed out that as long as one takes the recommended dosage, there is no risk from taking dietary supplements.

"If there is a very exaggerated consumption of certain supplements, but not something that I can say… Maybe anything that is taken too much, is bad, I guess, but I've never encountered that personally or heard of such a thing." (G., male trainer)

"Of consumption? So the risk comes only in high dosages. What is a high dosage? Twice the recommended dosage… If you take more than that, if they take it frequently, maybe twice the consumption, in that case it may happen, it may happen but it has to be examined on a case-by-case basis, I don't think that side effects come so fast because a person needs a little bit more than this supplement." (G., a male trainer)

"Side effects… I know about steroids, but I don't know much about dietary supplements." (A., a female trainer and body builder)

"There is a 100% benefit and it is better than taking medications…" (V., a male trainer)

#### Risk communication by trainers

Trainers were aware that the supplements might have side effects, but when asked about the benefits, they felt they exceeded this risk, and the risk was not perceived as a certainty. Trainers who were interviewed said that they do not warn gym members about side effects.

"No, we usually don't talk about it that much, I haven't heard or learned about such a supplement either but I always warn them and say: Look, I'm not the right professional to give you dosages and diets so you should read about it and learn about it by yourselves as well, if you decide about some supplement that you want to take – read about it, learn about it more deeply." (A., a female trainer and body builder)

#### Risk perception according to dietitians

The dietitians’ perspectives differed as they maintained that taking supplements involves risk and should be guided by professionals.

"I have a very harsh opinion about them, I think they are excellent for those who manufacture them but for us they are less good. I use supplements where it has clearly been proved that there's a certain deficit and only then do I give the body what it specifically requires, I prefer to have a doctor or pharmacist looking at the dosages, I don't make light of the supplements." (Y., a clinical dietitian and sports dietitian)

"There are side effects; as for creatine, for example, there's a phenomenon of water accumulation while taking it and it hurts your flexibility and you have to watch out for injuries, or, iron supplements have side effects to your digestion system… There are always side effects that we should pay attention to." (M., a clinical dietitian and fitness trainer)

#### Risk communication by dietitians

In the interviews, the dietitians reported that they also warn their clients about some of the side effects and health risks that the supplements can carry and teach the subject in certification courses for fitness instructors and trainers.

"Always, and I also ask them to take blood tests. I want to have an indication. I will ask. For example, omega 3 supplements cause clotting disorders, just like curcumin in sports dietary supplements. There might also be kidney problems and liver problems as a result of protein supplements. In general, I'm cautious, particularly about kidney and liver functions." (H., a dietitian)

### Sources of information and reliability

Based on the interviews, most gym members obtain information about dietary supplements from information channels on social networks and from friends, but less from fitness instructors and dietitians. Gym members who use SDS determine the reliability of a supplement according to its rating on talk shows and websites:

"…If the supplement is highly rated then it is more moderated, I probably take what the majority of people take." (S., a female trainee, an MA student from Singapore)

"…I simply read the list of ingredients, and then I try to get an idea of each one. I have common sense; I take product A and compare it to a product of manufacturer B, which is supposed to do the same thing and apparently comes from the same kind of protein…" (Y., a male trainee, a student)

#### Uncertainty about information reliability

Although users seem to examine the reliability of information they read on the internet according to criteria they decide on, such as ratings on talk show and websites, when they were asked about supplement reliability, their responses revealed uncertainty:

"Good question. I assume that a large part of it is marketing." (A., male gym member)

"The product is sold according to basic marketing principles, and in my opinion that's how it works as well. But that doesn't mean that it doesn't help at all, though the description of the product is not real in my opinion, their promises…" (H., a male trainee, a broker)

"…People come to a gym so they see certain instructors and notice that someone takes supplements, and then they're like, 'Maybe I should take them too', many times people ask me, "Do I also have to take this?" So where do they get this idea from? Because you hear from your friends, that guy takes this, so you say, maybe I'm wrong that I'm not taking this too." (M, a female trainee, a rugby player)

### Impact of social norms on supplement consumption

Most gym members who took sports dietary supplements had been exposed to people who took such supplements, such as friends and family members, thereby normalizing supplement consumption.

"…In my family everybody's old already so… I’m just kidding, but I remember two uncles of mine who were weightlifters. When they came to visit, the first thing they took out of their suitcase were dietary supplements. In my family, everybody works out or used to work out. That's just one more family affair, like jobs, money…" (R., a female gym member)

#### Impact of physical environment on sports dietary supplement consumption

According to most interviewees, it is legitimate for the gym to sell sports dietary supplements. Most gym members know where they are located.

"I think it's good, because if you forget to bring them with you or you want to buy them here… They also have shakes…" (M., an American female gym member)

"I think it's good that it's here. If you haven't eaten adequately and you want to train, so you still can do that…" (S., a Singaporean female gym member)

A number of reservations were raised by some of the gym members regarding the accessibility of the products, when they are not accompanied by explanations from professionals. They did not have reservations about their presence at the gym:

"I think it's perfectly fine but it has to be accompanied by someone who explains what… If it’s necessary, how much and in what way and for whom it is right and in what dosages. It has to be a part of a process." (M., a female gym member)

We found that the prevailing opinion among interviewees was that selling supplements at the gym might influence gym members to consume them. However, some said that their availability did not affect them directly. Trainers’ opinions about selling supplements at the gym were also positive. The trainers said that the sales boosted income for trainers and for the gym. The trainers also noted the gym members’ need to obtain the supplements. According to the trainers, by selling supplements at the gym the gym did the members a service:

"As for trainers, if they have the ability to make money out of it, then they'll try, and it happens in many chain sports clubs. The members think they need it, because he sees it and thinks this might help him a little bit. And we're in such a society of here and now and want to get our thing." (G., a male trainer)

The dietitians interviewed for this study reprehended selling supplements at the gym. Some appreciated the marketing and business aspect, but disagreed with unmonitored selling and said that trainers tended to encourage supplement purchase.

"In my opinion this is something that should be criticized. I know it's a part of the 2016 business in sports clubs. Large chain sports clubs earn their living off it, but I think that's the wrong step to take… I wouldn't have left the decision to provide dietary supplements in the trainers' hands but rather in more qualified hands… This is not what's actually happening…" (G. a clinical dietitian and education professional)

### Influence of authority

Gym members were asked who influenced them to take sports dietary supplements: a trainer, a dietitian or a doctor. Although interviewees said that the dietitian, physician and even trainer were authoritative, they also relied on the internet and on friends.

Some said that the trainer was available and accessible and therefore was the information source. Some said that the dietitian or the doctor were reliable sources, and some said that trainers and dietitians knew more about dietary supplements than the doctor. Doctors are not perceived as the foremost authorities on this subject:

"Of the three, the most authoritative person is the one who is in the same place as the gym member. He will have the strongest connection to him. The gym member will trust him because he is most available. 'Here I'm working out now - come and give me some advice.' But if he goes to a doctor and wants to ask him this is also fine. But you usually ask whoever is accessible … " (M. a female gym member)

## Discussion

People who work out at the gym constitute a central target for the dietary supplement market [45]. Although the literature discusses the prevalence of SDS use among athletes, it rarely discusses and compares between the risk perceptions of SDS by gym members, trainers and dietitians.

SDS are sold in many gyms and sports clubs, displayed and made accessible. This reinforces the normativity of supplement use.

Most gym members do not consult professionals before buying these products but rather other members or trainers, making consumption uncontrolled and sometimes injudicious.

In addition, the interviews in this study indicated that trainers are aware that the SDS might have side effects. However, according to the interviews, the benefit exceeds the risk, and the risk is perceived as uncertain. Therefore, they do not raise the issue of side effects and do not communicate the risk to gym members. The dietitians were clear about the risk of supplement use and the need for a dietitian to direct them by providing reliable nutritional information. Some of them claimed that taking SDS at the recommended dosage is not hazardous, but it is not necessarily beneficial. All the dietitians who participated in this study said that people should be cautious about taking SDS unnecessarily, and that they communicate this risk both to gym members and to those studying to be trainers. The gap between perceptions of gym members, trainers and dietitians makes the risk communication to gym members difficult, based on Sandman’s model [34]. Sandman notes that when there is a gap between experts’ and the public’s risk perception and severity, it is hard to communicate risk to the public. In this case the study found that gym members’ risk perception regarding taking SDS was low (low hazard level) and most of them were not concerned about taking SDS (low outrage level), as opposed to the dietitians – the experts in this case – who were concerned by the risk (high outrage level) and perceived the hazard to be high.

The interviews with the gym members and trainers also indicated that they learned about SDS through social networks and friends. The current study found that gym members and trainers develop criteria according to which they determine reliability of SDS and information on the internet. The study indicates a congruence between the findings and the social model of the “theory of planned behavior” (TPB), which suggests that an individual will tend to take action when there is a combination of subjective norms with the belief that the action is controlled and taken out of choice [46].

A study on dietary supplements, which examined the communication between doctors and patients, found that patients tended to take supplements without consulting with their doctors due to their belief that doctors are not knowledgeable about this [47]. The present study may shed light on the interaction with gym members who take SDS without consulting professionals due to their mistrust of the information. The FDA, WHO and NIH recommend that the public consult with health experts prior to taking dietary supplements, and that the experts must conduct a preliminary inquiry into supplement use, a discussion of its safety and effectiveness, and follow up on its effects [48]. The findings of the present study reveal that uncertainty perceived by gym members can be explained by gaps in professional guidance and lack of regulation and law enforcement for supplement advertising and marketing.

## Conclusion

The findings of this study facilitate an understanding of why dietary supplement use is so widespread. It shows that there is a gap in the risk perception between trainers, dietitians and gym members regarding this issue. This study can constitute a basis for recommendations for the Health Ministry and for sports associations to increase the importance of apprising gym members about the uncertainty and about the importance of judicious consumption, while providing clear information about supplements.

### Study limitations

Due to the qualitative nature of this study, it is not capable of representing and generalizing about research sub-populations. However, this study enables us to look more deeply into the phenomenon of consumers and the perspectives of different sub-populations regarding an understudied subject. Additionally, the participants in this study actually use sports dietary supplements and are healthy, so their perceptions are influenced by this. In this research, the personal bias of the dietitians and trainers regarding SDS use (such as conflict of interest due to personal benefits they may receive following their SDS use recommendations), nor their background knowledge, education and training has been explored. We suggest that future research will explore the impact, if any, of these variables on the way dietitians and trainers inform and consult gym members on SDS use. Finally, we suggest that more research should investigate different sub populations that are “healthy” and “unhealthy” that use SDS supplements.

#### Recommendations

Future studies can add perspectives of additional populations who work out. Most consumers in this study lacked knowledge about supplement side effects, and further research should ascertain whether risk communication might affect participants’ awareness of risks. This study also indicates that the information about SDS is gleaned particularly from the internet and friends; further studies should research the distribution of information on the internet.

## Abbreviations

DAA: Dimethylamylamine
DSHEA: Dietary Supplement Health and Education Act
FDA: Food and Drug Administration
NIH: National Institutes of Health
TPB: Theory of Planned Behavior
WHO: World Health Organization
